# Regulator of G protein signaling 16 restrains apoptosis in colorectal cancer through disrupting TRAF6-TAB2-TAK1-JNK/p38 MAPK signaling

**DOI:** 10.1038/s41419-024-06803-6

**Published:** 2024-06-21

**Authors:** Hao Shen, Jie Yuan, Dafeng Tong, Bingchen Chen, Enda Yu, Guanglei Chen, Cheng Peng, Wenjun Chang, Jifu E, Fuao Cao

**Affiliations:** 1 Department of Colorectal Surgery, The First Affiliated Hospital of Naval Medical University, Shanghai, China; 2 Department of Environmental and Occupational Health, Naval Medical University, Shanghai, China; 3 Department of Health Management, Beidaihe Rest and Recuperation Center of PLA Joint Logistics Support Force, Qinhuangdao, China

**Keywords:** Colorectal cancer, Tumour biomarkers, Prognostic markers

## Abstract

Colorectal cancer (CRC) remains a major global cause of cancer-related mortality, lacking effective biomarkers and therapeutic targets. Revealing the critical pathogenic factors of CRC and the underlying mechanisms would offer potential therapeutic strategies for clinical application. G protein signaling (RGS) protein family modulators play essential role within regulating downstream signaling of GPCR receptors, with function in cancers unclear. Our study focused on the expression patterns of RGS proteins in CRC, identifying Regulator of G protein signaling 16 (RGS16) as a prospective diagnostic and therapeutic target. Analyzing 899 CRC tissues revealed elevated RGS16 levels, correlating with clinicopathological features and CRC prognosis by immunohistochemistry (IHC) combined with microarray. We confirmed the elevated RGS16 protein level in CRC, and found that patients with RGS16-high tumors exhibited decreased disease-specific survival (DSS) and disease-free survival (DFS) compared to those with low RGS16 expression. Functional assays demonstrated that RGS16 promoted the CRC progression, knockdown of RGS16 led to significantly increased apoptosis rates of CRC in vitro and in vivo. Notably, we also confirmed these phenotypes of RGS16 in organoids originated from resected primary human CRC tissues. Mechanistically, RGS16 restrained JNK/P38-mediated apoptosis in CRC cells through disrupting the recruitment of TAB2/TAK1 to TRAF6. This study provides insights into addressing the challenges posed by CRC, offering avenues for clinical translation.

## Introduction

Colorectal cancer (CRC), constituting a highly prevalent malignancy, serves as a significant contributor to global morbidity and mortality. Notably, CRC occupies the second position in terms of cancer-related mortality on global scale, it also stands third in terms of incidence rate among all malignancies [1]. Currently, surgical intervention stands as the cornerstone in the treatment of early-stage CRC patients, achieving a commendable 5-year survival rate exceeding 90%. This success is attributed to advancements in surgical techniques and conventional modalities like radiation, chemotherapy, and neoadjuvant therapy. However, for individuals afflicted with stage IV colon and rectal cancers, the 5-year survival rate plummets to a mere 11% and 15%, respectively [2–4]. Therefore, the identification of innovative and distinctive molecules capable of accurately prognosticating CRC outcomes, along with the exploration of novel therapeutic targets, assumes paramount importance in mitigating the mortality burden associated with this ailment.

Evading cell death is widely recognized as a hallmark of cancer [5]. Apoptotic cell death is essential for the limitation of tumor growth. Nevertheless, the resistance to apoptosis, arising from dysregulated expression of molecules involved in its regulation and mediation, represents a pivotal step in tumor progression [6]. Therefore, elucidating the oncogenic targets, unraveling the molecular processes underlying tumor cells’ resistance to apoptosis can offer novel insights into tumorigenesis and therapeutic approaches.

The regulator of G protein signaling (RGS) protein family, characterized by a conserved RGS homology (RH) domain [7], interacts with G protein subunits α (Gα), playing a crucial role in regulating downstream pathways of GPCRs by enhancing GTP hydrolysis from Gα [8]. GPCRs have been extensively implicated in oncogenic processes [9], emphasizing the potential of targeting RGS proteins involved in the GPCR pathway for cancer therapy [10]. RGS16 is characterized by a conserved RH domain and an α-helix [11], serving as a member of the small B/R4 subfamily of RGS proteins. Expression of RGS16 has been reported to vary across multiple tumor types such as breast cancer [12], pancreatic cancer tissue [13], neuroblastoma [14], and others, either increasing or decreasing, suggesting its unique functional role. Nevertheless, the precise involvement of RGS16 in CRC remains a subject of debate. Buckbinder et al. propose that RGS16as a P53-sensitive gene, exhibits significant expression in RKO cells, potentially acting as a tumor suppressor during malignancy progression, its role in CRC remains contentious [15]. Conversely, another study showed opposing conclusion that strong RGS16 expression in CRC patients correlates with worse overall survival (OS) rates [16]. Consequently, further elucidation is warranted to determine the specific functions of RGS16 in CRC, including its impact on tumor staging and its biological role within CRC cells. In this comprehensive study, we conducted a large-scale clinical analysis, identifying CRC patients with elevated RGS16 expression. Moreover, through experimental investigations, we delineated the oncogenic function of RGS16, demonstrating its crucial involvement in promoting resistance to apoptosis in CRC, thus establishing it as a therapeutic target for CRC.

## Materials and methods

### Data mining in TCGA

RNA sequencing (RNA-seq) data from 695 CRC patients, as well as clinical data from 627 CRC individuals, were obtained from The Cancer Genome Atlas (TCGA) database (https://portal.gdc.cancer.gov) up to December 12, 2022. Samples without significant clinicopathological or lacking survival information were excluded. Given the freely accessible nature of TCGA data, the study was deemed exempt from approval by local ethics boards. The entirety of this study followed the TCGA data access regulations and publication standards.

The scale approach from the “limma” R package was employed to standardize gene expression profiles. K-M survival analysis was conducted on the TCGA datasets by the “survival” R package to investigate the link between RGS genes abundance and cancer outcome, specifically OS and progression-free survival (PFS). The outcomes of the survival analysis were calculated by the “survival” R package and visualized by the “forestploter” R package. Association between molecular patterns and clinical characteristics was investigated using the “limma” R package. Clinical variables encompassed age, gender, and pathological stage.

### Patients and follow-up

In this investigation, a cohort comprising 899 subjects experiencing primary lesion resection for CRC between 2010 and 2017 were assembled from the Department of Colorectal Surgery at the First Affiliated Hospital of Naval Medical University, Shanghai, China. 899 tumor specimens, 38 adjacent normal tissues (situated at a distance within 5 cm from the tumor edge), 37 normal tissues, and 16 liver metastasis specimens were enrolled.

The demographic particulars of all patients, including their name, gender, hospitalization number, wax block number, and operation date, alongside their clinical and pathological data (tumor location, differentiation grade, postoperative adjuvant treatment, TNM staging, preoperative serum CEA, and preoperative serum CA199), were encompassed from the hospital’s medical record system. The TNM stage of each patient was determined based on the NCCN guidelines (2021 edition). Follow-up assessments were conducted every 6 months to document recurrence, metastasis, and the survival status of each patient. Missing data were excluded. Follow-up information was collected from subjects with stage I–IV CRC following the previously described standard. Primary research outcomes during this study period were recorded as disease-specific survival (DSS) and disease-free survival (DFS). In compliance with the Helsinki Declaration principles, every individual supplied signed consent with knowledge.

### Cell lines

Cell lines CCD-18CO, RKO, Caco-2, and SW480 applied in the research were supplied by Shanghai Institute of Life Sciences, a constituent of Chinese Academy of Sciences. The short tandem repeat sequences of these cell lines were identified by Suzhou Kuisai Biotechnology Co. Ltd. and subsequently archived in the primary laboratory of First Affiliated Hospital of Naval Medical University. RPMI 1640 medium with addition of 10% fetal bovine serum (FBS) (10099141C, Gibco, Grand Island, NY, USA), 25 mM HEPES (22400-089, Gibco, Grand Island, NY, USA), 2 mM L-glutamine was employed for aforementioned CRC cell line cultivation and kept at 37 °C in a moist condition, with 5% CO_2_.

### Intestinal crypts, adenomas, and CRC-derived organoids’ culture

Isolated and cultured according to established protocols, organoids originating from human colon crypts, adenomas, and CRC samples were obtained [17, 18]. The tissue samples were finely sliced into small 5 mm^3^ fragments with surgical scissors in a cell culture dish. Colonic tissue was thoroughly rinsed with precooled DPBS until the supernatant became clear. The sheared tissue was resuspended in a solution of primary tissue digest (MB-0818L05S, ABW, Shanghai, China) at 50 times the tissue volume and subjected to digestion with horizontal shaking for 30 min at 37 °C in a constant temperature shaker set at 100 rpm. FBS (10099141C, Gibco, Grand Island, USA) was supplemented to the digested tissue suspension at a final concentration of 2–5%, to alleviate the digestive effect. The above tissue suspension was filtered through a 100 μm cell filter. The mixture was centrifuged at 250 × *g* at 4 °C for 3 min, the supernatant was aspirated, and the precipitate was retained. Cells were resuspended in basal medium containing antibiotics (MA-0817H001S, ABW, Shanghai, China), and extracellular matrix (>70%) (082755, ABW, Shanghai, China) was added at 100,000–500,000 cells/ml, then mixed on ice. The mixed cells were added to a 24-well cell culture plate (3473, CORNING, New York, USA), 20–30 μl of the mixed suspension was used for each well spot. The cell culture plate was positioned within incubator for the culture process. The growth of CRC organoids was observed under an inverted microscope every day, photos were taken under an inverted microscope every 2 days to record the morphology and distribution of organoids in multiple fields.

### siRNA transfection and lentiviral transfection

Three pairs of siRNAs were designed to target consensus sequences of RGS16 transcript variants, namely siRNA1 (5’ - GAT CTT TCT TCA CAA ATC A - 3’), siRNA2 (5’ - GGA GTA CTG GCA AGT TCG A - 3’), and siRNA3 (5’ - ACG CTT TCC TGA AGA CAG A - 3’). The design of these siRNAs was outsourced to GenePharma Co. Ltd. (Shanghai, China), utilizing a concentration of 20 nM for the transfection process. The control siRNA-seq used was 5’ - UUC UCC GAA CGU GUC ACG UTT - 3’, representing a nonfunctional sequence. Transient transfection of the target genes was carried out following the prescribed instructions using the Lipofectamine RNAiMAX transfection kit (13778150, ThermoFisher, Waltham, USA). For quantifying mRNA expression through quantitative PCR analysis, the ChamQ SYBR qPCR Master Mix (Q311-02, Vazyme, Nanjing, China) was employed. These siRNAs were generated employing analogous principles used for coding genes.

The lentivirus packing procedure was overseen by Shanghai Heyuan Biotechnology Co. Ltd., located in Shanghai, China. Table S1 provides a comprehensive listing of the specific sequences utilized to construct lentiviral vectors to overexpress or knockdown RGS16 in CRC cell lines.

For stable target gene expression, CRC cell lines and organoids underwent infection with the respective viruses, and subsequent selection using puromycin (Solarbio, Shanghai, China).

### Mice and antibodies

BALB/c nude mice (♂), 6–8 weeks old, were procured from Joint Ventures Sipper BK Experimental Animal (Shanghai, China). All experiments using animals adhered strictly to the National Institutes of Health Guide for the Care and Use of Laboratory Animals and were sanctioned by the Scientific Investigation Board of Navy Military Medical University, Shanghai. Specific antibodies targeting RGS16 (ab119424), TAK1 (ab109526), p-TAK1 (ab109404), TRAF6 (ab40675), GAPDH (ab8245), p38 (ab170099), p-p38 (ab178867), JNK (ab179461), p-JNK (ab124956), Cleaved-caspase 3 (ab32042), and Cleaved-PARP (ab32064) were procured from Abcam, Cambridge in UK and employed, so did TAB2 (AF4635, Affinity, OH, USA). Takinib (EDHS-206) was obtained from MedChemExpress (Monmouth, NJ).

### IHC staining

Immunohistochemistry staining was performed as described [19] to determine the expression level of RGS 16 in CRC. IHC was also employed for the detection of ki67 and cleaved caspase 3 expression in CRC organoids and tumor xenografts in mice.

### Multiplex immunohistochemical staining in fluorescence

Multiplex immunohistochemical staining in fluorescence are detailed in the Supplementary Materials.

### RNA quantification

RNA quantifications are described in detail in the Supplementary Materials.

### WB and CoIP

The details of the experimental methods regarding WB and CoIP can be found in the Supplementary materials.

### Cell growth, migration, invasion and apoptosis assays

Migration, invasion, apoptosis assays, clone formation, and cell viability for CRC cells in vitro were studied as the statements in the Supplementary Materials.

### In vivo xenograft model

The animal experiments detailed in this report strictly adhered to the ethical guidelines outlined in the “National Health Department Animal Experiment Management Regulations,” which provide the ethical framework for animal research in China. Approval to conduct these experiments was obtained from the Scientific Investigation Committee of the Naval Medical University of Shanghai, responsible for overseeing and regulating animal experimentation within the university. During the experiment, Caco-2 cells were subcutaneously inserted into the axillas of mice, and mice with subcutaneous lesions meeting the specified mass parameters (70–100 mm^3^) were selected for further examination. To minimize potential bias in the data, the experimental settings were kept confidential and undisclosed to the technicians responsible for monitoring tumor size.

### RNA-sequencing and data analysis

The procedures for RNA-sequencing (RNA-seq) and subsequent data analyses are thoroughly described in the Supporting Information.

### Statistical analysis

The statistical software packages R 4.1.3 and GraphPad Prism 8.0 were utilized for the creation of all visualizations and statistical analyses. Clinical data were presented as count data cases (*n*) and subjected to analysis using the chi-square test. The disparities in OS, PFS, DFS, and DSS among subgroups were computed employing the Kaplan–Meier method and expressed as HR with 95% CI estimated through the normal distribution. We applied COX proportional hazard model was in both univariate and multivariate analyses for specific prognostic variable impacts. Statistical differences between two samples or cohorts were assessed by either Mann–Whitney *U*-test or Student’s *t* test. Analysis of variance was employed for comparing means across multiple cohorts. Significance: *p* < 0.05.

## Results

### The expression level of RGS16 is particularly abundant in CRC and correlate with 5-year survival rate

**Fig. 1:**
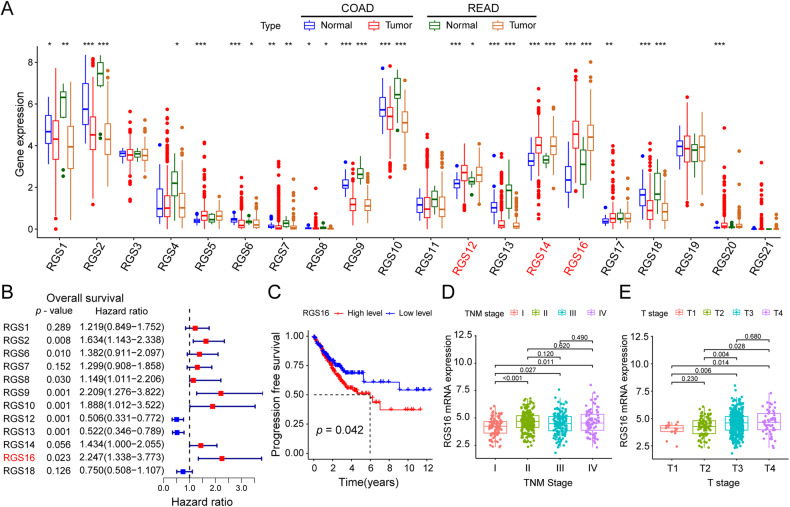
Association between the expression of RGS16 with prognosis and clinical characteristics of CRC in the TCGA database. **A** Expression difference of RGS family genes in tumor tissues and normal tissues in the COAD and READ program of TCGA database. Among these genes, RGS12, RGS14, and RGS16 demonstrated elevated expression levels in CRC tissues, while RGS1, RGS2, RGS6, RGS7, RGS8, RGS9, RGS10, RGS13, and RGS18 exhibited low expression in such tissues. **B** RGS16 was the unique family member that is highly expressed in tumor tissue and held predictive significance in terms of OS. **C** Kaplan–Meier curves showing differences in the progression free survival of patients in the TCGA database stratified according to their mRNA expression levels of RGS16 using the median cutoff (*n* = 627, log-rank test). **D**, **E** There were differences in pathological stage (**D**) and T stage (**E**) between high expression group and low expression group divided into according to the median expression of RGS16 as the cut-off value. *, **, and *** indicate that *p* is below 0.05, 0.01, and 0.001, respectively.

We conducted a comprehensive examination of the expression patterns of RGS proteins in colon and rectum adenocarcinoma (COAD and READ) utilizing the TCGA database. Subsequently, we identified 12 highly promising candidates (RGS1/2/6/7/8/9/10/12/13/14/16/18) that exhibited distinct expression profiles between malignant and adjacent normal tissues in both COAD and READ (Fig. 1A). Among these candidates, RGS12, RGS14, and RGS16 exhibited elevated expression levels in CRC tissues, while the remaining RGS proteins displayed low expression in such tissues (Fig. 1A). Notably, RGS16 emerged as the unique family member with high expression in tumor tissue, holding predictive significance for both OS (Fig. 1B) and PFS (Fig. 1C). In comparison, other family members exhibited less significance than RGS16 concerning expression difference and prognostic value (Supplementary Fig. S1A, B). Further exploration in TCGA databases unveiled a correlation between RGS16 expression and the extent of TNM stage classification (stage I–stage IV) (Fig. 1D) as well as initial tumor invasion (T1–T4) (Fig. 1E).

**Fig. 2:**
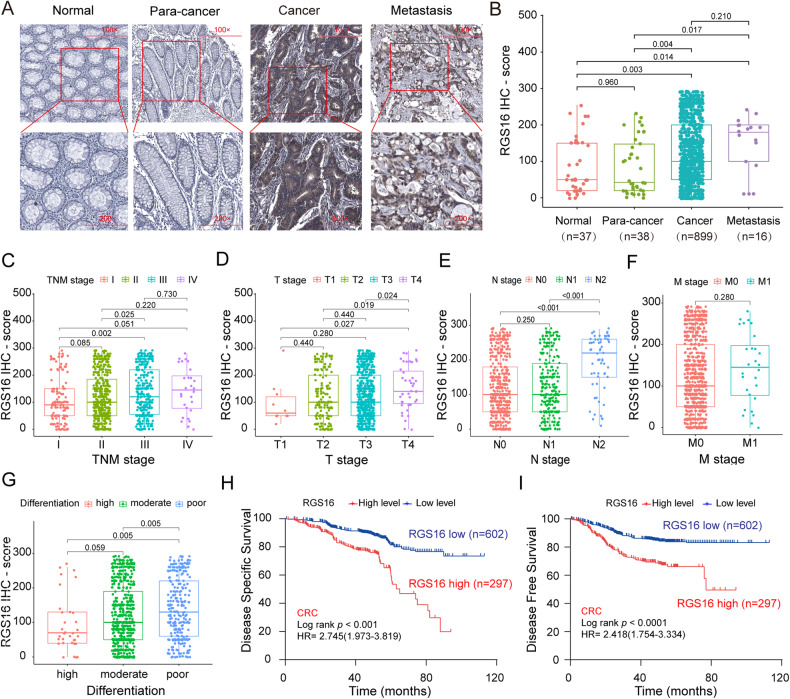
Expression of RGS16 in CRC tissues and association with prognosis in CRC TMAs. **A** Illustrative examples of immunostaining for RGS16 protein expression in various tissue types including normal tissues, para-cancer tissues, cancer tissues and metastasis tissues. Original magnifications, ×100 and ×200. **B** IHC scores of RGS16 protein expression in TMAs showed that the expression of RGS16 increased significantly from normal tissues to adjacent tissues and then to cancer tissues and metastasis tissues (*p* < 0.05). **C**–**F** Elevated RGS16 expression was associated with pathological stage (**C**), T stage (**D**), and N stage (**E**), but not with distant metastasis (**F**). **G** The expression of RGS16 in tumor tissues is related to the differentiation grade. **H**, **I** Kaplan–Meier survival analysis showing DSS (**H**) and DFS (**I**) in 899 CRC patients from TMAs, separated based on the best cutoff value of RGS16 IHC scores (*p* < 0.001).

Through the utilization of a tissue microarray encompassing 37 normal colorectal tissues, 38 colorectal para-cancer tissues, 899 CRC tissues, and 16 metastatic CRC samples, along with an IHC analysis to discern the expression of RGS16 protein, we conducted a screening of positive signals and computed the IHC score to quantify the degree of RGS16 expression in each tissue sample. Our investigation revealed that, in comparison to the adjacent tissues, CRC tissues and metastatic CRC tissues exhibited significantly higher levels of RGS16 protein, predominantly localized within the cytoplasm (*p* < 0.05; as depicted in Fig. 2A, B). Moreover, we found that elevated RGS16 expression was associated with tumor stage (Fig. 2C), T stage (Fig. 2D), N stage (Fig. 2E), and grade of differentiation (Fig. 2G), but not with distant metastasis (Fig. 2F). Furthermore, we examined the association between RGS16 protein levels and the clinical outcomes of CRC patients. Notably, patients displaying elevated RGS16 expression (IHC score >150) exhibited a shorter DSS (2.745, 95% CI: 1.973–3.819) and DFS (2.418, 95% CI: 1.754–3.334) in contrast to those with lower RGS16 expression (IHC score ≤150) (Fig. 2H, I).

**Fig. 3:**
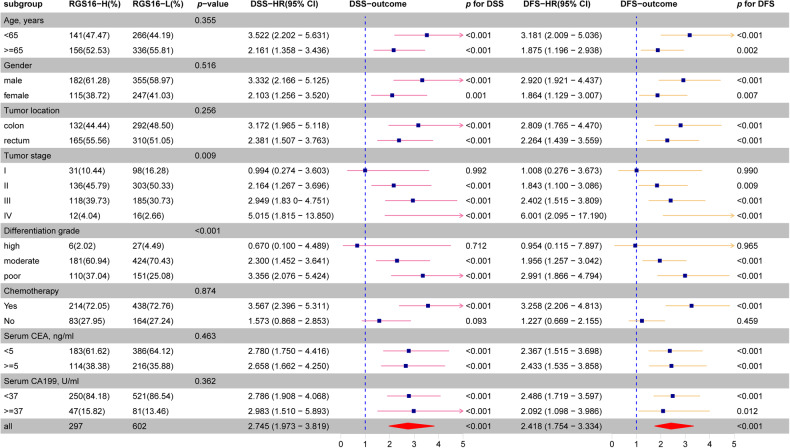
Correlation between RGS16 expression and clinicopathological parameters and survival analysis of RGS16 in different subgroups of colorectal cancer patients. The patients with CRC were categorized into distinct subgroups based on age, gender, tumor location, pathological stage, degree of differentiation, adjuvant chemotherapy administration, and serum tumor markers. The prognostic value of RGS16 was found to be more remarkable in patients with advanced stage, or moderately–poorly differentiation, or those undergoing adjuvant chemotherapy.

During the conduction of survival analysis within distinct subsets of age, gender, tumor location, TNM stages, differentiation grades, chemotherapy, and serum tumor markers, a remarkable inverse correlation was observed between heightened expression of RGS16 and DFS/DSS among patients diagnosed with stage II–IV CRC, patients undergoing adjuvant chemotherapy as well as moderately to poorly differentiated CRC patients (Fig. 3 and Supplementary Fig. S2A–R). These data imply that RGS16 is potentially indicative of CRC prognosis and play substantial role in CRC progression.

Based on Cox analysis result, an elevated expression of RGS16 exhibited a significant association with CRC patients DFS and DSS in the univariate Cox analysis (as shown in Table 1). Moreover, factors such as RGS16 expression, differentiation grade, TNM stage, and serum biomarkers were found to be linked with DFS and DSS of CRC patients. Importantly, after adjusting for grade, stage, serum CEA, and serum CA199, multivariate Cox regression analysis demonstrated that increased RGS16 expression in CRC served as a statistically significant predictor of DFS (2.278, 95% CI: 1.693–3.067) and DSS (2.615, 95% CI: 1.937–3.530).

**Table 1:** Cox regression analysis of immunohistochemistry RGS16 expression and clinicopathological covariates in the CRC patients.

Variables		Disease-specific survival				Disease-free survival			
		Univariate analysis		Multivariate analysis		Univariate analysis		Multivariate analysis	
		HR (95% CI)	*p* value	HR (95% CI)	*p* value	HR (95% CI)	*p* value	HR (95% CI)	*p* value
Age, years	<60	Ref	Ref			Ref	Ref		
	≥60	0.904 (0.675–1.211)	0.500			0.875 (0.653–1.172)	0.369		
Gender	Female	Ref	Ref			Ref	Ref		
	Male	0.987 (0.734–1.328)	0.933			0.949 (0.706–1.277)	0.732		
Tumor location	Colon	Ref	Ref			Ref	Ref		
	Rectum	1.018 (0.757–1.369)	0.906			0.856 (0.638–1.147)	0.297		
Tumor stage	I	Ref	Ref	Ref	Ref	Ref	Ref	Ref	Ref
	II	0.601 (0.446–0.811)	**<0.001**	1.090 (0.589–2.016)	0.784	0.631 (0.468–0.851)	**0.003**	1.309 (0.708–2.419)	0.390
	III	1.648 (1.228–2.213)	**<0.001**	1.702 (0.912–3.176)	0.095	1.668 (1.242–2.239)	**0.001**	2.061 (1.106–3.843)	**0.023**
	IV	2.613 (1.581–4.318)	**<0.001**	2.661 (1.220–5.804)	**0.014**	3.881 (2.353–6.400)	**<0.001**	4.830 (2.216–10.531)	**<0.001**
Differentiation	High	Ref	Ref	Ref	Ref	Ref	Ref	Ref	Ref
	Moderate	0.608 (0.453–0.816)	**<0.001**	0.653 (0.280–1.522)	0.323	0.548 (0.408–0.735)	**<0.001**	0.602 (0.258–1.405)	0.241
	Poor	1.680 (1.247–2.263)	**<0.001**	0.834 (0.348–1.995)	0.683	1.898 (1.410–2.553)	**<0.001**	0.845 (0.353–2.024)	0.706
Chemotherapy	No	Ref	Ref	Ref	Ref	Ref	Ref	Ref	Ref
	Yes	0.730 (0.533–1.000)	0.050			0.784 (0.573–1.074)	0.130		
Serum CEA, ng/ml	<5	Ref	Ref	Ref	Ref	Ref	Ref	Ref	Ref
	≥5	1.709 (1.275–2.291)	**<0.001**	1.359 (0.991–1.863)	0.057	1.753 (1.308–2.349)	**<0.001**	1.36 (0.991–1.867)	0.057
Serum CA199, U/ml	<37	Ref	Ref	Ref	Ref	Ref	Ref	Ref	Ref
	≥37	2.043 (1.449–2.879)	**<0.001**	1.452 (0.997–2.115)	0.052	2.218 (1.574–3.125)	**<0.001**	1.498 (1.027–2.184)	**0.036**
RGS16, IHC score	≤150	Ref	Ref	Ref	Ref	Ref	Ref	Ref	Ref
	>150	2.836 (2.105–3.820)	**<0.001**	2.615 (1.937–3.530)	**<0.001**	2.438 (1.817–3.270)	**<0.001**	2.278 (1.693–3.067)	**<0.001**

Bold values indicate statistical significance *p* < 0.05. In the multivariate model, only variables with *p* < 0.05 in univariate analysis were included.

*HR* hazard ratio, *95% CI* 95% confidence interval.

### RGS16 augmented the viability, invasion, and migration of CRC cells

**Fig. 4:**
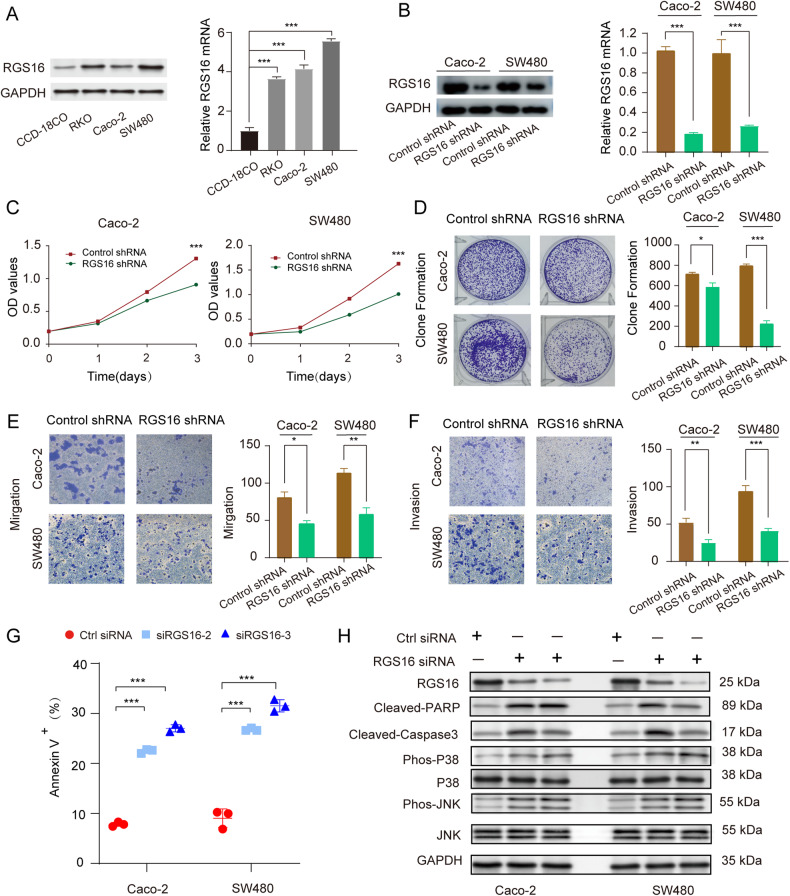
Knocking down RGS16 attenuates CRC cell progression in vitro. **A** The protein and mRNA levels of RGS16 in human normal colon cell line (CCD-18CO) and CRC cell lines (RKO, SW480 and Caco-2) were detected by western blot and qRT-PCR, respectively. **B** WB and qRT-PCR were used to measure the protein and mRNA concentrations of RGS16 in Caco-2 and SW480 cells that had been stably transfected with either a control (Ctrl) or shRGS16 vector. **C** Cell viability of Caco-2 and SW480 cells transfected with the Ctrl or shRGS16 vector was determined using the CCK-8 assay at 1, 2, and 3 days to measure cell proliferation. **D** Representative images of SW480 and Caco-2 cells after lentivirus infection were captured after 12 days of culture, and the number of cells was analyzed in a clonal growth formation experiment. **E**, **F** Migration and invasion of SW480 and Caco-2 infected cells were assessed using transwell membranes, as depicted in representative images. Quantitative analysis of migrated and invaded Caco-2 and SW480 cells was conducted. **G** Caco-2 and SW480 cells were transfected with either a control siRNA or two RGS16 siRNAs (20 nM each) for 48 h. Apoptotic cells positive for Annexin V were quantified using the Annexin V/PI assay. The average standard deviation of triple samples from an appropriate experiment is included in the data presented. **H** After 48 h of transfection, SW480 and Caco-2 cells were treated with either Ctrl or RGS16 siRNA (RGS16 RNAi #2 and #3). WB analysis was conducted to examine the indicated proteins. The data represent the mean ± SD of at least three independent experiments. The *p* values in (**A**, **G**) were calculated by one-way ANOVA. The *p* values in (**B**–**F**) were calculated by Student’s *t* test. *, **, and *** indicate that *p* is below 0.05, 0.01, and 0.001, respectively.

After analyzing clinical samples, we proceeded to investigate the regulatory functions of RGS16 in CRC cells. Notably, both RGS16 protein and mRNA concentrations in CRC cell lines surpassed normal colonic fibroblast line CCD-18CO (Fig. 4A). Subsequently, we conducted cell viability (CCK8 assay), colony formation, cell migration, and invasion experiments in CRC cell lines (Caco-2 and SW480 cells) that were infected with a lentivirus containing RGS16-specific shRNA. To begin with, we verified the successful knockdown of RGS16 mRNA and protein levels in Caco-2 and SW480 cell lines using the shRNA targeting RGS16 (Fig. 4B). The CCK8 and colony formation assays revealed a substantial reduction in cell viability (Fig. 4C) and colony formation capacity (Fig. 4D) upon RGS16 knockdown in CRC cells in comparison to the control group. Furthermore, by employing the transwell technique with or without Matrigel, knockdown RGS16 significantly hampered CRC cells’ mobile and aggressive capabilities (Fig. 4E, F). Additionally, we successfully overexpressed RGS16 in CRC cells (Supplementary Fig. S3A) to further validate its role, and observed that it significantly promoted clone formation, infiltration, proliferation, and metastasis in CRC cells (Supplementary Fig. S3B–E). These findings strongly support the oncogenic activity of RGS16, highlighting that increased expression of RGS16 enhances the infiltration, proliferation, viability, and metastasis of CRC cells.

### RGS16 restrained JNK/p38 MAPK-mediated apoptosis of CRC cell

Apoptosis, recognized as a pivotal regulator of cellular viability, was explored to investigate the impact of RGS16 on CRC cell through the utilization of flow cytometry analysis coupled with Annexin V labeling. The outcomes suggested a considerable elevation in Annexin V-positive CRC cells rate after RGS16 knockdown, demonstrating that RGS16 depletion triggered apoptosis in CRC cells (Fig. 4G). Hence, it is postulated that RGS16 may function as an anti-apoptotic protein within the context of CRC. For further research into the mechanisms underlying RGS16-mediated protection against cell death in CRC, we examined the signaling pathways associated with apoptosis subsequent to RGS16 knockdown. Our investigation revealed an elevation in caspase 3 and PARP cleavage, alongside an upregulation in the phosphorylated levels of JNK and p38 MAPK following RGS16 depletion (Fig. 4H). Outcomes align with prior research indicating JNK/p38 MAPK activation promotes apoptosis in tumor cells via enhancing the cleavage of caspase 3 and PARP [20]. Consequently, it can be inferred that RGS16 modulates apoptosis in CRC cells by impeding JNK/p38 MAPK activation and the ensuing cleavage of caspase 3 and PARP.

### Knockdown RGS16 inhibits CRC progression in vivo

**Fig. 5:**
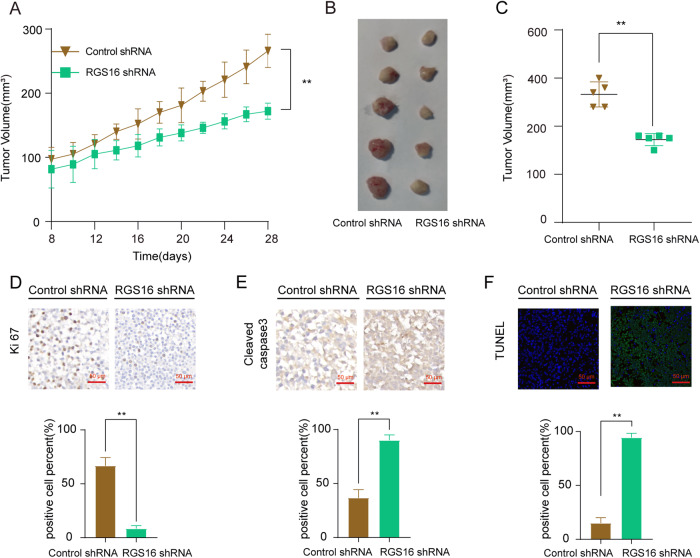
The impact of RGS16 on CRC cells in vivo. **A** Subcutaneous inoculation of mice was performed using Caco-2 cells that had been stably transfected with vectors, including Ctrl and shRGS16. Tumor volume was measured as indicated when it reached 70–100 mm^3^ (Day 8) and calculated as *V* = (width^2^ × length)/2. **B** After 4 weeks of feeding, the subcutaneous graft tumor volume in the RGS16 knockdown group was smaller than that in the control group. **C** The morphology of tumor xenografts from each nude mouse was photographed. **D**, **E** Immunohistochemistry staining of slices from xenografts was used to detect the expression of Ki67 (**D**) and cleaved caspase 3 (**E**). Scale bars, 50 μm. **F** TUNEL staining of tumor xenografts from the above two groups are shown. Scale bars, 50 μm. The data represent the mean ± SD of at least three independent experiments. The *p* values in panle were calculated by Student’s *t* test. ** indicate that *p* is below 0.01. *n* = 5 mice/group.

To substantiate the in vivo implications of RGS16, CRC mouse model was established employing CRC cells that were stably transfected with either RGS16 knockdown (RGS16 shRNA) or overexpression plasmid (Flag RGS16). Our investigation revealed that RGS16 overexpression significantly augmented tumor burden (Supplementary Fig. 4A–C), whereas RGS16 knockdown markedly curtailed tumor growth in vivo (Fig. 5A–C). The Ki67 labeling of tumor tissue depicted a positive correlation between RGS16 and tumor cell proliferation activity, whereas the Tunel assay and immunohistochemistry staining of cleaved caspase 3 exhibited negative association between RGS16 and apoptosis levels in tumor cells (Fig. 5D–F and Supplementary Fig. S4D–F). Consequently, our findings provide compelling evidence suggesting that RGS16 fosters tumor growth by impeding tumor cell apoptosis in an in vivo setting.

**Fig. 6:**
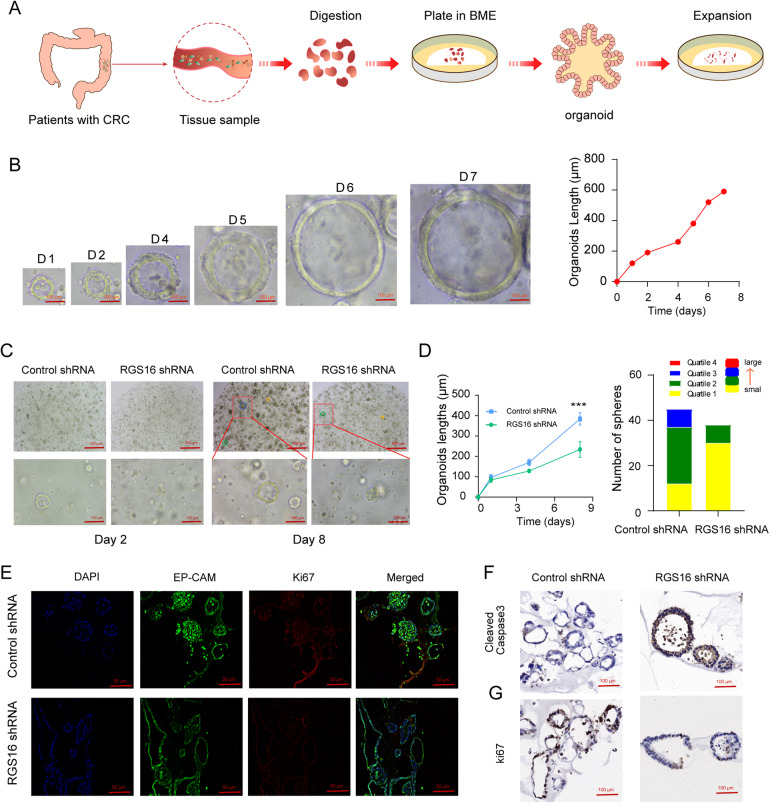
Knocking down RGS16 facilitated apoptosis of tumor cell in CRC organoids. **A** Schematic description of the organoids development. **B** The representative graph showed the culture of CRC organoids from Day 1 to Day 7. The length of organoids was recorded by line chart. Scale bars, 100 μm. **C** Images represented the CRC organoids after transfection with Ctrl and shRGS16 vectors at Day 2 and Day 8. CRC organoid length and sphere numbers of the above two groups was recorded. **D** The length of organoids in RGS16 knockdown group was smaller than that in control group. The proportion of smaller organoids in the knockdown group was also higher than that in the control group. **E** Multiplex fluorescent immunohistochemistry staining images of organoids expressing specific markers Ki67 (red), EP-CAM (green) with blue DAPI staining. **F**, **G** The representative graphs depicted the IHC staining of the aforementioned two groups of CRC organoids to illustrate Cleaved Caspase 3 (**F**) and ki67 (**G**) expression levels in both groups of organoids. The data represent the mean ± SD of at least three independent experiments. The *p* values were calculated by Student’s *t* test. *** indicate that *p* is below 0.001.

To further elucidate the impact of RGS16 in CRC, organoids were generated from resected primary human CRC tissues (Fig. 6A). To evaluate organoid growth, images were captured daily for a week following resuscitation (Fig. 6B). We knocked down RGS16 by using the shRNA targeting RGS16 which was packaged into lentivirus (Supplementary Fig. 5A). During the sphere growth period, RGS16 knocking down significantly inhibit the organoids size than that of control organoids (Fig. 6C). We also assessed the spheres of the organoids. The sub-histogram visually represents organoid area distributions in quartiles. In this representation, the red and green colors depict the 25th and 75th quartiles, respectively, of the distribution, with organoid areas increasing. Colored area length corresponds to spheres count within the specified size range. Our result indicated that the organoid areas was much smaller in RGS16 knockdown group than that in control group (Fig. 6D). For a more comprehensive assessment of CRC organoids, immunohistochemical multiplex fluorescent IHC analysis demonstrated epithelial cell adhesion molecule (EpCAM) expressions and CRC biomarker Ki67. Notably, high Ki67 staining indicated elevated proliferative activity (Fig. 6E). Immunohistochemical results suggested that RGS16 could inhibit the apoptosis while promote the proliferation of CRC organoids, which further suggested that RGS16 potentially promote CRC progression (Fig. 6F, G). In addition, the overexpression of RGS16 in CRC organoids was further verified as Supplementary Fig. S5B, and we observed that RGS16 significantly promoted the growth of CRC organoids (Supplementary Fig. 5C–F).

### RGS16 disrupt TRAF6/TAB2/TAK1 complex generation

**Fig. 7:**
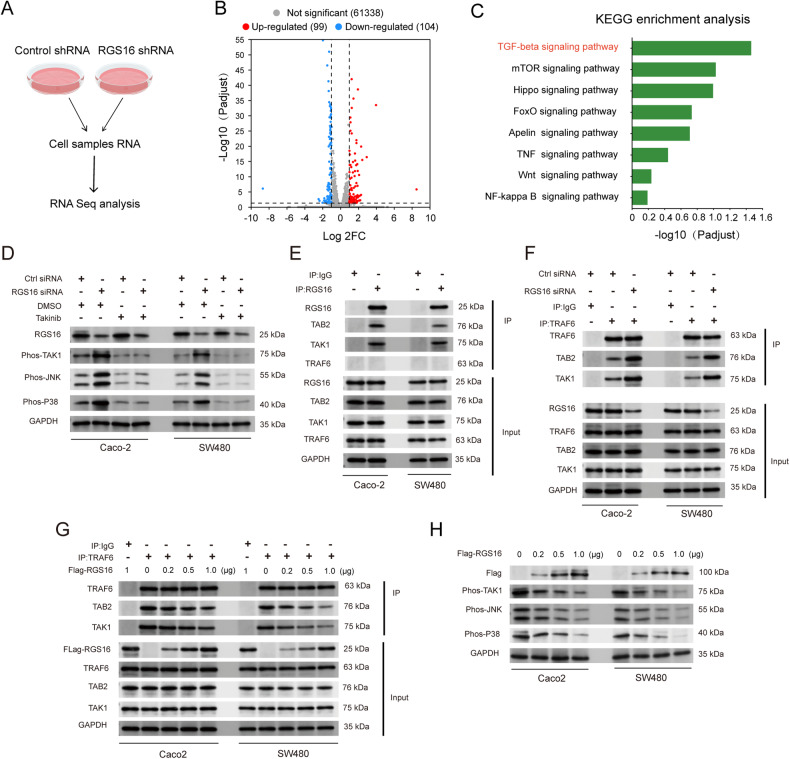
RGS16 disrupt TRAF6-TAB2 interaction and inhibit TAK1-JNK/p38 MAPK activation. **A** RNA-seq was performed on Caco-2 cells transfected with Ctrl and shRGS16 vectors. **B** Volcano map showing DEGs in above two groups. The red dots represent up-regulated DEGs, and the blue dots represent down-regulated DEGs. The gray dots represent genes without significant differences in expression. **C** KEGG enrichment analysis was performed between SW480 cells transfected with RGS16 shRNA vector and the control group. **D** the Caco-2 and SW480 cells were transfected with control and RGS16 siRNA for 48 h, and then treated with DMSO or Takinib for 8 h. The RGS16 and the activation of indicated protein were examined by Western blotting. **E** The whole cell extracts (input) were immunoprecipitated with anti-IgG or anti-RGS16 antibody plus protein A/G beads. Components in the RGS16 complex were examined by Western blotting. **F** The Caco-2 and SW480 cells were transfected with control and RGS16 siRNA for 48 h. Then the whole cell extracts (input) were immunoprecipitated with anti-IgG or anti-TRAF6 antibody plus protein A/G beads. Components in the TRAF6 complex were examined by Western blotting. **G** The Caco-2 and SW480 cells were transfected with Flag-RGS16 as indicated for 48 h. Then the whole cell extracts (input) were immunoprecipitated with anti-IgG or anti-TRAF6 antibody plus protein A/G beads. Components in the TRAF6 complex were examined by Western blotting. **H** The Caco-2 and SW480 cells were transfected with Flag-RGS16 as indicated for 48 h. The RGS16 and the activation of indicated protein were examined by Western blotting. One representative experiment of three is shown.

**Fig. 8:**
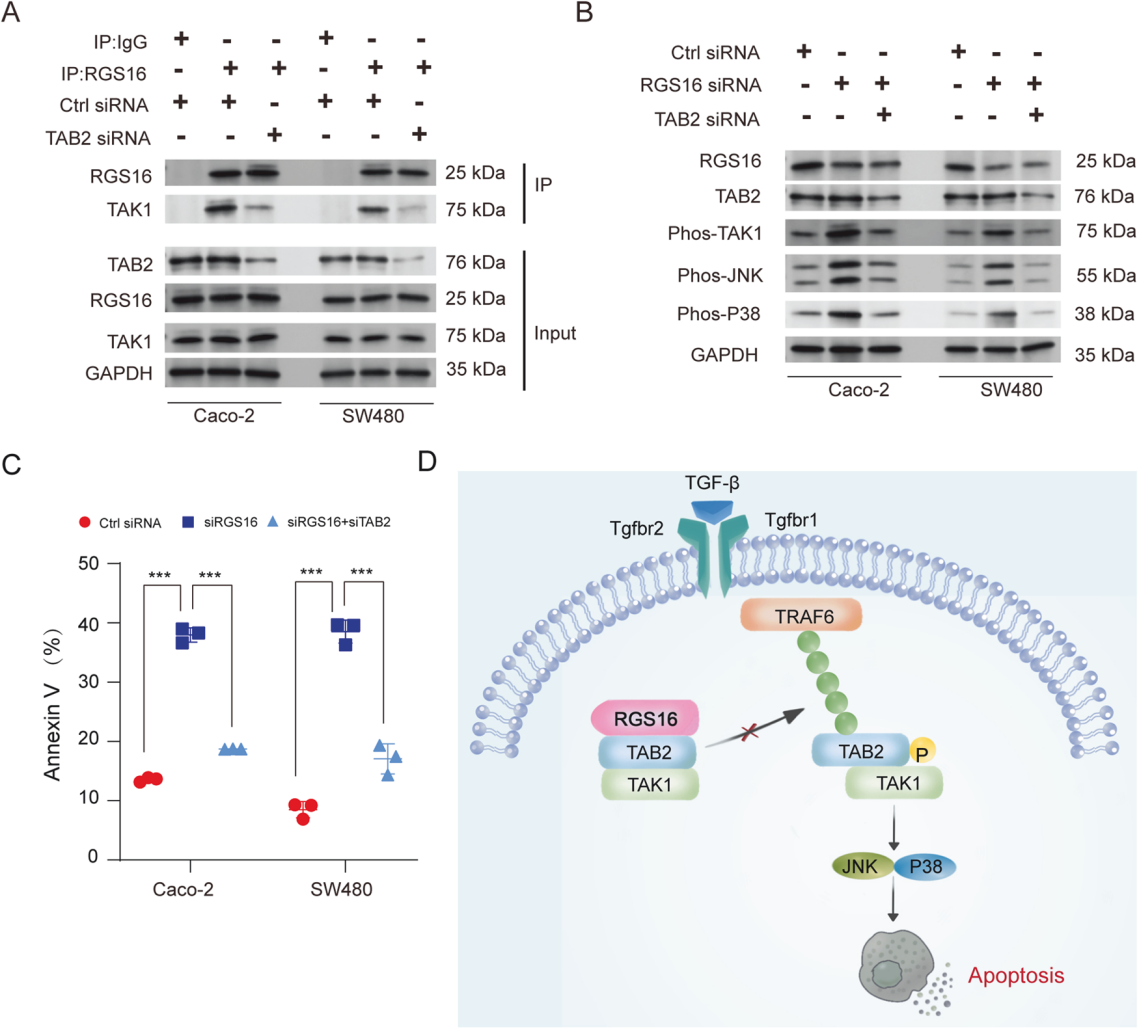
TAB2 is required for the RGS16 knockdown-mediated enhancement of TAK1-JNK/p38 MAPK-induced apoptosis. **A** The Caco-2 and SW480 cells were transfected with control and TAB2 siRNA for 48 h. Then the whole cell extracts (input) were immunoprecipitated with anti-IgG or anti-RGS16 antibody plus protein A/G beads. Components in the RGS16 complex were examined by Western blotting. **B** The Caco-2 and SW480 cells were transfected with control, RGS16 and TAB2 siRNA as indicated for 48 h. The RGS16, TAB2 and the activation of indicated protein were examined by Western blotting. **C** The cell in (**B**) were stained with Annexin V/PI, then the percentage of Annexin V^+^ cells were tested by FACS. **D** Graph abstract of RGS16 in the regulation of cell apoptosis. One representative experiment of three is shown. Results are presented as mean ± SD of three biological replicates (**C**, one-way ANOVA followed by Bonferroni multiple comparison). *** indicates that *p* is below 0.001.

For the investigation on promotive effect mechanism by RGS16 on CRC progression, we performed RNA-seq using RGS16 knockdown cells (Fig. 7A). Volcano mapping provided additional confirmation that the majority of differentially expressed genes (DEGs; fold change >1, *p* < 0.05) were up-regulated (Fig. 7B). KEGG enrichment analysis in RNA-seq suggested the potential involvement of RGS16 in the TGF-β signaling pathway (Fig. 7C). Previous reports indicate that the non-canonical, non-Smad pathways of TGF-β signaling are activated directly by ligand-occupied receptors, regulating downstream cellular responses, including the TAK1/JNK/p38 MAPK pathway, which is pivotal for TGF-β-induced apoptosis [21]. We found that knockdown of RGS16 enhanced TAK1, JNK and p38 MAPK phosphorylation while the TAK1 inhibitor could reverse their activation (Fig. 7D). The immunoprecipitation result showed that, RGS16 formed a complex with TAB2 and TAK1 (Fig. 7E). TAB2 is a critical adapter by linking TAK1 to TbetaRI/TRAF6 [22]. We further found knockdown of RGS16 increased the interaction between TRAF6 and TAB2/TAK1 and RGS16 could dose dependently inhibit the recruitment of TAB2/TAK1 by TRAF6 (Fig. 7F, G). Functionally, RGS16 could also restrain TAK1, JNK and p38 MAPK activation dose dependently (Fig. 7H). Furthermore, TAB2 knockdown could weaken the interaction between RGS16 and TAK1 (Fig. 8A). And TAB2 knockdown could also rescue JNK/p38 MAPK activation and the subsequent apoptosis as well as the inhibitory effect of cell viability, migration, invasion and clonal formation of CRC cell which were caused by the interference of RGS16 (Fig. 8B, C and Supplementary Fig. 6). These results suggested that RGS16 inhibited TRAF6-TAB2-TAK1 complex formation and downstream JNK/p38 MAPK activation, which was dependent on its interaction with TAB2 (Fig. 8D).

## Discussion

CRC represents a substantial global menace, characterized by its elevated mortality rate and associated health complications. The absence of discernible clinical symptoms during the early stages hampers timely diagnosis, resulting in approximately 25% of CRC cases being identified with distant organ metastasis, thereby exacerbating prognosis and outcomes [23]. Presently, neoadjuvant chemotherapy, radiation, and immunotherapy constitute the three major therapeutic modalities employed for advanced CRC management [3]. In recent decades, numerous molecular-targeted agents, such as Epidermal Growth Factor Receptor (EGFR), Tropomyosin Receptor Kinase (TRK), Vascular Endothelial Growth Factor Receptor (VEGFR), and Anaplastic Lymphoma Kinase (ALK), have exhibited remarkable antitumor efficacy across various tumor types, including CRC [24–27]. However, due to the intricate pathophysiology of CRC, the impact of a solitary medication on CRC therapeutics remains limited. Combining diverse targeted drugs is envisaged to enhance the efficiency and efficacy of anticancer therapy for CRC. Nevertheless, this approach may give rise to amplified side effects and engender treatment resistance, necessitating exploration of novel therapeutic targets for CRC. Our investigation has identified the overexpression of RGS16 in CRC, which correlates positively with disease progression, implying its indispensability in disease advancement and rendering it a unique and prospective therapeutic target for CRC.

GPCR comprise the largest receptor family located on the cellular membrane surface. These receptors facilitate signal transmission to cells through G protein coupling, thereby governing numerous cellular functions [28]. The regulatory RGS protein serves as determinant in terminating GPCR signals by expediting the hydrolysis of the GTP enzyme. Consequently, dysregulation of RGS is correlated with various ailments, including cardiovascular and neurodegenerative disorders, as well as cancer [29, 30]. Significantly, abnormal expression of multiple RGS proteins, such as RGS17 in hepatocellular carcinoma, lung cancer, and prostate cancer, RGS5 in ovarian cancer and squamous cell carcinoma, and RGS6 in breast cancer and urinary bladder cancer, has been documented in relation to tumor progression [31–34]. In this study, we utilized a tissue microarray consisting of more than 899 clinical samples, covering the TNM stages and differentiation states of CRC. With comprehensive prognosis data available, we conducted a systematic exploration of the clinicopathological characteristics of RGS16. Our research revealed that RGS16 serves as a reliable prognostic marker for DFS and DSS in individuals with CRC, especially in those with advanced TNM stages or poor differentiation statuses. Hence, our findings suggest that RGS16 has the potential to be a distinctive clinic diagnostic biomarker for CRC.

The functional investigation has unveiled the capacity of RGS16 to potentiate the viability, migratory potential, and invasive properties of CRC cells, while concurrently impeding their susceptibility to apoptosis. The signal pathway analyses have implicated that RGS16 accomplishes this modulation by hampering JNK/p38 MAPK activation, consequently thwarting PARP and Caspase 3 cleavages. Hence, the available data strongly suggest that RGS16 exerts a promoting effect on CRC progression through the inhibition of apoptosis. Existing research indicates the indispensability of JNK activation in governing apoptosis in various malignancies, including gastric cancer [35–37]. JNK/p38 MAPK pathway activation occurs through a cascade involving MAP3Ks and MAP2Ks, encompassing ASK1-3, TAK1, MEKK1-4, MLK1-3, DLKs, MKK4, and MKK7 [38]. Our work demonstrated that RGS16 binds to TAB2 and inhibits TAB2-TRAF6 interaction thus exerted a suppressive effect on TAK1-JNK/p38-mediated apoptosis. The highly conserved zinc finger (ZnF) domain of TAB2 is known to mediate its binding to the polyubiquitin chain on TRAF6 [39]. We hypothesize that RGS16 may prevent TAB2 from binding to TRAF6 by interacting with the ZnF domain of TAB2. However, further research is necessary to validate our hypothesis.

The research of new therapeutic targets and methods for CRC has been the hot topic of cancer research. Among them, small molecule compounds that targeting novel CRC markers are one area where several progresses have been made [40–44]. In our study, RGS16, as a novel apoptosis suppressor in CRC, mainly inhibits the activation of TAK1-JNK/p38-mediated apoptosis signals through structural blocking. Therefore, if we select small molecule compounds that block RGS16’s binding to the TRAF6/TAB2 complex by screening the library of natural or synthetic small molecule compounds, we can develop novel potential therapies for CRC through targeting RGS16. On the other hand, gut microbiome plays a double-edged role in the progression of CRC [45]. Among the regulatory mechanisms, gut microbiome can also activate JNK/p38 signaling [46] which suggests that targeting RGS16 may enhance the apoptosis of CRC cells induced by gut microbiome.

In summary, our observations have demonstrated a noteworthy upregulation of RGS16 expression in CRC, alongside a negative association with TNM stage and the 5-year survival rate of CRC patients. Moreover, we have substantiated that RGS16 enhances the viability, migratory capacity, and invasive properties of CRC cells while concurrently suppressing their apoptotic tendencies in both in vitro and in vivo settings. Mechanistic investigations have further unveiled that RGS16 impedes JNK/p38 signaling activation by inhibiting TAB2/TAK1 to TRAF6 recruitment, thereby impeding the cleavage of PARP and the expression of Caspase 3 associated with apoptosis. The stimulatory role of RGS16 in regulating the viability of CRC cells suggests that RGS16 may serve as an oncogene driving tumor progression. Notably, our study has, for the first time, revealed the capacity of RGS16 to modulate JNK/p38-mediated apoptosis in CRC cells. In patients with CRC, RGS16 serves as a prognostic marker and predictor of tumor progression, potentially representing a future therapeutic target.

### Supplementary information

supplementary material

supplementary material

## Data Availability

The data generated in this study are available upon request from the corresponding author.

## References

[1] Sung H, Ferlay J, Siegel RL, Laversanne M, Soerjomataram I, Jemal A (2021). Global cancer statistics 2020: GLOBOCAN estimates of incidence and mortality worldwide for 36 cancers in 185 countries. CA Cancer J Clin.

[2] Kannarkatt J, Joseph J, Kurniali PC, Al-Janadi A, Hrinczenko B (2017). Adjuvant chemotherapy for stage II colon cancer: a clinical dilemma. J Oncol Pract.

[3] Miller KD, Nogueira L, Devasia T, Mariotto AB, Yabroff KR, Jemal A (2022). Cancer treatment and survivorship statistics, 2022. CA Cancer J Clin.

[4] Sonbol MB, Mountjoy LJ, Firwana B, Liu AJ, Almader-Douglas D, Mody K (2020). The role of maintenance strategies in metastatic colorectal cancer: a systematic review and network meta-analysis of randomized clinical trials. JAMA Oncol.

[5] Hanahan D, Weinberg RA (2011). Hallmarks of cancer: the next generation. Cell.

[6] Cao K, Tait SWG (2018). Apoptosis and cancer: force awakens, phantom menace, or both?. Int Rev Cell Mol Biol.

[7] Watson N, Linder ME, Druey KM, Kehrl JH, Blumer KJ (1996). RGS family members: GTPase-activating proteins for heterotrimeric G-protein alpha-subunits. Nature.

[8] Tesmer JJ, Berman DM, Gilman AG, Sprang SR (1997). Structure of RGS4 bound to AlF4–activated G(i alpha1): stabilization of the transition state for GTP hydrolysis. Cell.

[9] Wu V, Yeerna H, Nohata N, Chiou J, Harismendy O, Raimondi F (2019). Illuminating the Onco-GPCRome: novel G protein-coupled receptor-driven oncocrine networks and targets for cancer immunotherapy. J Biol Chem.

[10] O’Brien JB, Wilkinson JC, Roman DL (2019). Regulator of G-protein signaling (RGS) proteins as drug targets: progress and future potentials. J Biol Chem.

[11] Tian M, Ma Y, Li T, Wu N, Li J, Jia H (2022). Functions of regulators of G protein signaling 16 in immunity, inflammation, and other diseases. Front Mol Biosci.

[12] Hoshi Y, Endo K, Shirakihara T, Fukagawa A, Miyazawa K, Saitoh M (2016). The potential role of regulator of G-protein signaling 16 in cell motility mediated by δEF1 family proteins. FEBS Lett.

[13] Kim JH, Lee JY, Lee KT, Lee JK, Lee KH, Jang KT (2010). RGS16 and FosB underexpressed in pancreatic cancer with lymph node metastasis promote tumor progression. Tumour Biol.

[14] Liu T, Bohlken A, Kuljaca S, Lee M, Nguyen T, Smith S (2005). The retinoid anticancer signal: mechanisms of target gene regulation. Br J Cancer.

[15] Buckbinder L, Velasco-Miguel S, Chen Y, Xu N, Talbott R, Gelbert L (1997). The p53 tumor suppressor targets a novel regulator of G protein signaling. Proc Natl Acad Sci USA.

[16] Miyoshi N, Ishii H, Sekimoto M, Doki Y, Mori M (2009). RGS16 is a marker for prognosis in colorectal cancer. Ann Surg Oncol.

[17] Sato T, Stange DE, Ferrante M, Vries RG, Van Es JH, Van den Brink S (2011). Long-term expansion of epithelial organoids from human colon, adenoma, adenocarcinoma, and Barrett’s epithelium. Gastroenterology.

[18] Sato, van Es T, Snippert JH, Stange DE HJ, Vries RG, van den Born M (2011). Paneth cells constitute the niche for Lgr5 stem cells in intestinal crypts. Nature.

[19] Yuan Y, Qi G, Shen H, Guo A, Cao F, Zhu Y (2019). Clinical significance and biological function of WD repeat domain 54 as an oncogene in colorectal cancer. Int J Cancer.

[20] Guo F, Zhang C, Wang F, Zhang W, Shi X, Zhu Y (2020). Deubiquitinating enzyme USP33 restrains docetaxel-induced apoptosis via stabilising the phosphatase DUSP1 in prostate cancer. Cell Death Differ.

[21] Zhang YE (2009). Non-Smad pathways in TGF-beta signaling. Cell Res.

[22] Takaesu G, Kishida S, Hiyama A, Yamaguchi K, Shibuya H, Irie K (2000). TAB2, a novel adaptor protein, mediates activation of TAK1 MAPKKK by linking TAK1 to TRAF6 in the IL-1 signal transduction pathway. Mol Cell.

[23] van der Stok EP, Spaander MCW, Grünhagen DJ, Verhoef C, Kuipers EJ (2017). Surveillance after curative treatment for colorectal cancer. Nat Rev Clin Oncol.

[24] FDA approves regorafenib (Stivarga) for metastatic colorectal cancer. Oncology. 2012;26:896. 23175993

[25] Koumaki K, Kontogianni G, Kosmidou V, Pahitsa F, Kritsi E, Zervou M (2021). BRAF paradox breakers PLX8394, PLX7904 are more effective against BRAFV600Ε CRC cells compared with the BRAF inhibitor PLX4720 and shown by detailed pathway analysis. Biochim Biophys Acta Mol Basis Dis.

[26] Mullard A (2019). FDA notches up third tissue-agnostic cancer approval. Nat Rev Drug Discov.

[27] Valeri N (2019). Streamlining detection of fusion genes in colorectal cancer: having “faith” in precision oncology in the (tissue) “agnostic” era. Cancer Res.

[28] Syrovatkina V, Alegre KO, Dey R, Huang XY (2016). Regulation, signaling, and physiological functions of G-proteins. J Mol Biol.

[29] Alqinyah M, Hooks SB (2018). Regulating the regulators: epigenetic, transcriptional, and post-translational regulation of RGS proteins. Cell Signal.

[30] Koelle MR (1997). A new family of G-protein regulators - the RGS proteins. Curr Opin Cell Biol.

[31] Abe Y, Ogasawara S, Akiba J, Naito Y, Kondo R, Nakamura K (2019). Expression and role of regulator of G-protein signaling 5 in squamous cell carcinoma of the tongue. Clin Exp Dent Res.

[32] Maity B, Yang J, Huang J, Askeland RW, Bera S, Fisher RA (2011). Regulator of G protein signaling 6 (RGS6) induces apoptosis via a mitochondrial-dependent pathway not involving its GTPase-activating protein activity. J Biol Chem.

[33] Wang D, Xu Y, Feng L, Yin P, Song SS, Wu F (2019). RGS5 decreases the proliferation of human ovarian carcinoma‑derived primary endothelial cells through the MAPK/ERK signaling pathway in hypoxia. Oncol Rep.

[34] Yang J, Platt LT, Maity B, Ahlers KE, Luo Z, Lin Z (2016). RGS6 is an essential tumor suppressor that prevents bladder carcinogenesis by promoting p53 activation and DNMT1 downregulation. Oncotarget.

[35] Bai Y, Liu X, Qi X, Liu X, Peng F, Li H (2019). PDIA6 modulates apoptosis and autophagy of non-small cell lung cancer cells via the MAP4K1/JNK signaling pathway. EBioMedicine.

[36] Sun XL, Zhang XW, Zhai HJ, Zhang D, Ma SY (2020). Magnoflorine inhibits human gastric cancer progression by inducing autophagy, apoptosis and cell cycle arrest by JNK activation regulated by ROS. Biomed Pharmacother.

[37] Wagner EF, Nebreda AR (2009). Signal integration by JNK and p38 MAPK pathways in cancer development. Nat Rev Cancer.

[38] Ha J, Kang E, Seo J, Cho S. Phosphorylation dynamics of JNK signaling: effects of dual-specificity phosphatases (DUSPs) on the JNK pathway. Int J Mol Sci. 2019;20:6157. 10.3390/ijms20246157 PMC6941053 31817617

[39] Kanayama A, Seth RB, Sun L, Ea CK, Hong M, Shaito A (2004). TAB2 and TAB3 activate the NF-kappaB pathway through binding to polyubiquitin chains. Mol Cell.

[40] Fernández J, Silván B, Entrialgo-Cadierno R, Villar CJ, Capasso R, Uranga JA (2021). Antiproliferative and palliative activity of flavonoids in colorectal cancer. Biomed Pharmacother.

[41] Pagano E, Romano B, Cicia D, Iannotti FA, Venneri T, Lucariello G (2023). TRPM8 indicates poor prognosis in colorectal cancer patients and its pharmacological targeting reduces tumour growth in mice by inhibiting Wnt/β-catenin signalling. Br J Pharmacol.

[42] Romano B, Pagano E, Iannotti FA, Piscitelli F, Brancaleone V, Lucariello G (2022). N-acylethanolamine acid amidase (NAAA) is dysregulated in colorectal cancer patients and its inhibition reduces experimental cancer growth. Br J Pharmacol.

[43] Pagano E, Venneri T, Lucariello G, Cicia D, Brancaleone V, Nanì MF, et al. Palmitoylethanolamide reduces colon cancer cell proliferation and migration, influences tumor cell cycle and exerts in vivo chemopreventive effects. Cancers. 2021;13:1923. 10.3390/cancers13081923 PMC8073478 33923494

[44] Şahin T, Yılmaz B, Yeşilyurt N, Cicia D, Szymanowska A, Amero P (2023). Recent insights into the nutritional immunomodulation of cancer-related microRNAs. Phytother Res.

[45] Ağagündüz D, Cocozza E, Cemali Ö, Bayazıt AD, Nanì MF, Cerqua I (2023). Understanding the role of the gut microbiome in gastrointestinal cancer: a review. Front Pharmacol.

[46] Zhang X, Liu W, Zhang S, Wang J, Yang X, Wang R (2022). Wei-Tong-Xin ameliorates functional dyspepsia via inactivating TLR4/MyD88 by regulating gut microbial structure and metabolites. Phytomedicine.

