# Supplementary material for: Regulator of G protein signaling 16 restrains apoptosis in colorectal cancer through disrupting TRAF6-TAB2-TAK1-JNK/p38 MAPK signaling
Source: Cell Death Dis. 2024 Jun 21;15(6):438. doi: 10.1038/s41419-024-06803-6 (PMC11192724; doi:10.1038/s41419-024-06803-6)
Supplement: Supplementary file 1 — supplementary material [file 41419_2024_6803_MOESM1_ESM.pdf]

SUPPLEMENTARY MATERIALS

CCK8 Assays

Caco-2 and SW480 cell viability was evaluated by CCK-8 test kit (CK04, Dojindo, Japan). Transfected cells and their corresponding negative control counterparts were seeded in 96-well plates. Subsequently, CCK-8 solution 10 μl was added, mixture was incubated for 1 hour. Cell viability was determined from day 1 to day 3 by microplate reader at a wavelength of 450 nm.

Clone Formation Assays

1000 Caco-2 and SW480 transfected cells were planted into each well of a 6-well plate that contained 2 ml of growth media. The cells were then maintained in fresh media every 2 to 3 days for a period of 14 days after being twice rinsed with PBS. Following the incubation period, the cell colonies had been resolved and subjected to crystal violet staining. The quantity of cell colonies present in each well was determined and visualized using Graphpad Prism 8.0 software.

Invasion and Migration Assays

To initiate the procedure, extract the Matrigel gel from BD company, which has been stored at -80°C, and transfer it to an icebox. Once the gel reaches a liquid state, dilute the Matrigel gel with a dilution ratio of 1:8. Using a pre-cooled pipette tip, carefully draw up 100 μl of the diluted matrix gel and gently deposit it onto the bottom membrane of the Transwell chamber. After placing the Transwell chamber in a cell culture box, allow the basement membrane to undergo hydration before proceeding to the next step; note that this step does not involve migration. For the preparation of the cell suspension, it is recommended to subject the cells to 12-24 hours of serum deprivation before preparing the suspension. Afterward, digest the cells and resuspend them in serum-free culture medium, adjusting the cell concentration in the resuspension to 5×10^5/ml. Introduce the cells into the chamber by gently dispensing 100 μl of the serum-free cell suspension in a vertical manner using a pipette tip. Add 700 μl of complete culture medium (20% FBS culture medium) beneath the cell culture plate. Subsequently, place the Transwell plate in the incubator and allow it to remain there for 24 hours. For cell fixation and staining, carefully remove the Transwell chamber, aspirate the medium within the chamber, and perform two washes with PBS. Fix the cells by incubating them in a 4% paraformaldehyde solution for 15 minutes. After removing the fixing solution, stain the cells with crystal violet for 15 minutes. Gently wipe the cells in the Transwell chamber's top chamber using a clean cotton swab. For statistical analysis, select nine fields under a microscope to observe the stained cells and determine their count. Capture images and retain them for further analysis.

Flow Cytometer（FCM）

Transfected cells were seeded in 6-well plates at 5×10^5 cells. Subsequently, the cells underwent staining with 5 μl Annexin V-FITC and propidium iodide (PI) in 500 μl of buffer. Following a 10-minute incubation, the cells were washed twice with PBS. Sh-Control cells were utilized as the control group. Annexin V/PI apoptosis kit (BD, USA) was employed for the assessment of cellular apoptosis, and the obtained results were analyzed using an Agilent Flow Cytometer.

RNA Extraction and Quantitative Analysis

Use 1 ml of Trizol reagent per 10 cm^2 of surface area to swiftly introduce the solution into the culture plate and initiate cell lysis. Perform repeated pipetting for thorough mixing of the solution. It is crucial to determine Trizol quantity based on culture plate surface area, rather than the cell count, to avoid DNA contamination during RNA extraction. Centrifuge the cell suspension to collect the cells, and then add 1×10^7 cells to 1 ml of Trizol, using repeated pipetting. Do not wash the cells before Trizol addition to prevent mRNA degradation. Vigorously shake the mixture for 15 seconds after adding 0.2 ml of chloroform per 1 ml of Trizol. Let the mixture stand undisturbed (room temperature, 3 minutes). Post centrifugation, the sample will separate into three layers: an intermediate layer, a colorless aqueous phase (containing the majority of RNA), and a yellow organic phase at the bottom. Transfer the tube containing the aqueous phase, while the organic phase may be retained for subsequent DNA and protein separation processes. Precipitate the RNA by adding isopropanol. For each 1 ml of Trizol used, add 0.5 ml of isopropanol and incubate (room temperature, 10 minutes). Although RNA precipitation may not be visible prior to centrifugation, a gelatinous precipitate will become apparent on the bottom and sides of the tube after centrifugation. Remove the supernatant. Rinse the RNA precipitate with 75% ethanol. Add no less than 1 ml of 75% ethanol for every 1 ml of Trizol used. Discard the supernatant after centrifuging. Dry the RNA precipitate at room temperature or under vacuum for approximately 5 to 10 minutes. Dissolve the RNA by adding RNase-free water or 0.5% SDS, performing multiple pipetting steps for dissolution. Maintain the temperature between 55 and 60°C for approximately 10 minutes. Note that an SDS solution should not be used if the RNA is intended for enzyme digestion reactions. Store the dissolved RNA at -70°C in deionized formamide.

RNA-sequencing and data analysis

Conducting a rigorous molecular exploration, Shanghai Majorbio Bio-pharm Biotechnology Co., Ltd. (Shanghai, China) meticulously executed the critical steps of RNA purification, reverse transcription, library construction, and sequencing, all in strict adherence to the meticulous guidelines set forth by Illumina (San Diego, CA). The standardized Illumina® Stranded mRNA Prep and Ligation protocol were implemented, utilizing 1μg of total RNA to meticulously craft the RNA-seq transcriptome library. This intricate process encompassed the precise size selection of libraries for cDNA target fragments of 300 bp on a 2% Low Range Ultra Agarose gel, followed by a precisely executed PCR amplification stage using Phusion DNA polymerase (NEB) over a meticulously controlled 15 PCR cycles. The resulting library, meticulously quantified using Qubit 4.0, underwent detailed paired-end RNA-seq sequencing on the NovaSeq 6000 sequencer, yielding sequences of 2 × 150 bp read length. For the nuanced analysis of differential gene expression, the highly regarded DESeq2 tool was brought into play, imposing stringent criteria by considering DEGs with $|\log_2 FC| \geq 2$ and $P_{adjust} \leq 0.05$ as unequivocally significant DEGs.

Multiplex Immunohistochemical Staining in Fluorescence

Sections of formalin-fixed paraffin-embedded CRC organoids, sliced at 5 μm, were placed onto slides. The slides underwent a 2-hour baking period at 65 °C and duplicate immersion in xylene for 30 minutes. Subsequently, the slides were sequentially immersed

in 100%, 95%, and 75% ethanol, followed by a 3-minute immersion in water. Antigen retrieval was then carried out using sodium citrate (pH 6.0). Endogenous peroxidase blockers were used to inhibit endogenous peroxidase, while non-specific staining was blocked by 10% normal sheep serum. The primary antibody KI67 (1:50) (27309-1-AP, Proteintech, Chicago, USA) was added in drop and incubated (for the whole night, 4°C) in a humid chamber. After removal of chamber, the samples were rewarmed to room temperature, and then washed with PBST. Subsequently, CD326 (EpCAM) Monoclonal Antibody (1B7), Alexa Fluor™ 488, eBioscience™ (53-9326-42, Thermo, Waltham, USA) was applied at a working concentration of 1:50, and the secondary antibody F(ab')2 Fragment (Alexa Fluor® 594 Conjugate) configured with secondary antibodies (#8890, CST, Boston, USA) was used at a working concentration of 1:500. The mixture was well-mixed and incubated at 37°C in light-shielding environment for 1 hour. Slices got finely rinsed with PBST (0.05% Tween 20) 3 times for 10 minutes each. Staining procedure included incubation with DAPI in the dark at 37°C for 10 minutes, followed by washing sections with PBST (0.05% Tween 20) 3 times for 10 minutes each. Excess water was removed from the slices, sealed with glycerin, and stored away from light at 4°C.

RNA quantification

Cellular total RNA was extracted using Trizol (Thermo, Waltham, USA), and reverse transcription was completed by ReverTra Ace qPCR RT Kit (Toyobo, Osaka, Japan). Detailed RNA extraction techniques are provided in the supplementary data. Quantitative PCR analyses were accomplished by ChamQ SYBR qPCR Master Mix (Vazyme, Nanjing, China) for mRNA expression quantification. Primer sequences were as follows: 5′-CCA CGC TTT CCT GAA GAC-3′(forward) and 5′-GCC TCA CTG CAA ATG AAC T(reverse). Human GAPDH served as an internal control with the primers 5′-GGA GCG AGA TCC CTC CAA AAT-3′(forward) and 5′-GGC TGT TGT CAT ACT TCT CAT GG-3′ (reverse).

WB and CoIP

Protein extraction was completed by either general kit or nuclear and cytoplasmic protein extraction kit, followed by WB analysis.

We measured the protein concentrations by BCA assay kit (Beyotime Biotechnology, China). Identical amounts of protein were isolated via SDS-PAGE and electrophoretically transferred to PVDF membranes (Millipore, USA; ISEQ00010). GAPDH-specific antibodies (Abcam, Cambridge, UK; ab8245) were utilized as loading controls. HRP-conjugated secondary antibodies were applied, and protein signals were visualized using ECL detection reagents (Millipore, USA; WBKLS0500). For Co-immunoprecipitation (CoIP) experiments, total cell lysates were initially incubated with antibodies specific to RGS16 and TRAF6. To facilitate the formation of antibody-protein complexes, protein G-Sepharose beads from Thermo Fisher Scientific were introduced and allowed to bind to the lysate. To ensure specificity, the beads were subjected to three consecutive washes using lysis buffer, effectively removing non-specific interactions.The immunoprecipitated complexes were then eluted by boiling the beads for 5 minutes in SDS loading buffer. This elution process aimed to release the captured proteins from the antibody-bound beads. The eluted samples were subsequently analyzed using Western blotting techniques, following the established protocol mentioned earlier.

Table S1 Sequences of lentiviral vectors to overexpress or knockdown RGS16 in CRC cell lines

No.	Virus vector	Titer(TU/ml)
GL427NC2	pSLenti-U6-shRNA(NC2)-CMV-EGFP-F2A-Puro-WPRE	1.36E+09
Y20547	pSLenti-U6-shRNA(RGS16)-CMV-EGFP-F2A-Puro-WPRE	3.50E+08
GL180	pSLenti-EF1-EGFP-P2A-Puro-CMV-MCS-3xFLAG-WPRE	1.30E+08
H25436	pSLenti-EF1-EGFP-P2A-Puro-CMV-RGS16-3xFLAG-WPRE	1.90E+08

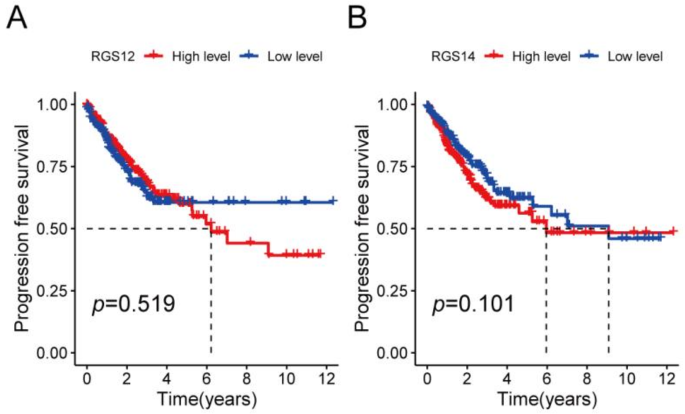

Supplementary Fig.S1 Kaplan-Meier curves of RGS family members. A, B Kaplan-Meier curves showing differences in the progression free survival of patients stratified according to their mRNA expression levels of RGS12(**A**) and RGS14(**B**) using the median cutoff (n = 627, log-rank test).

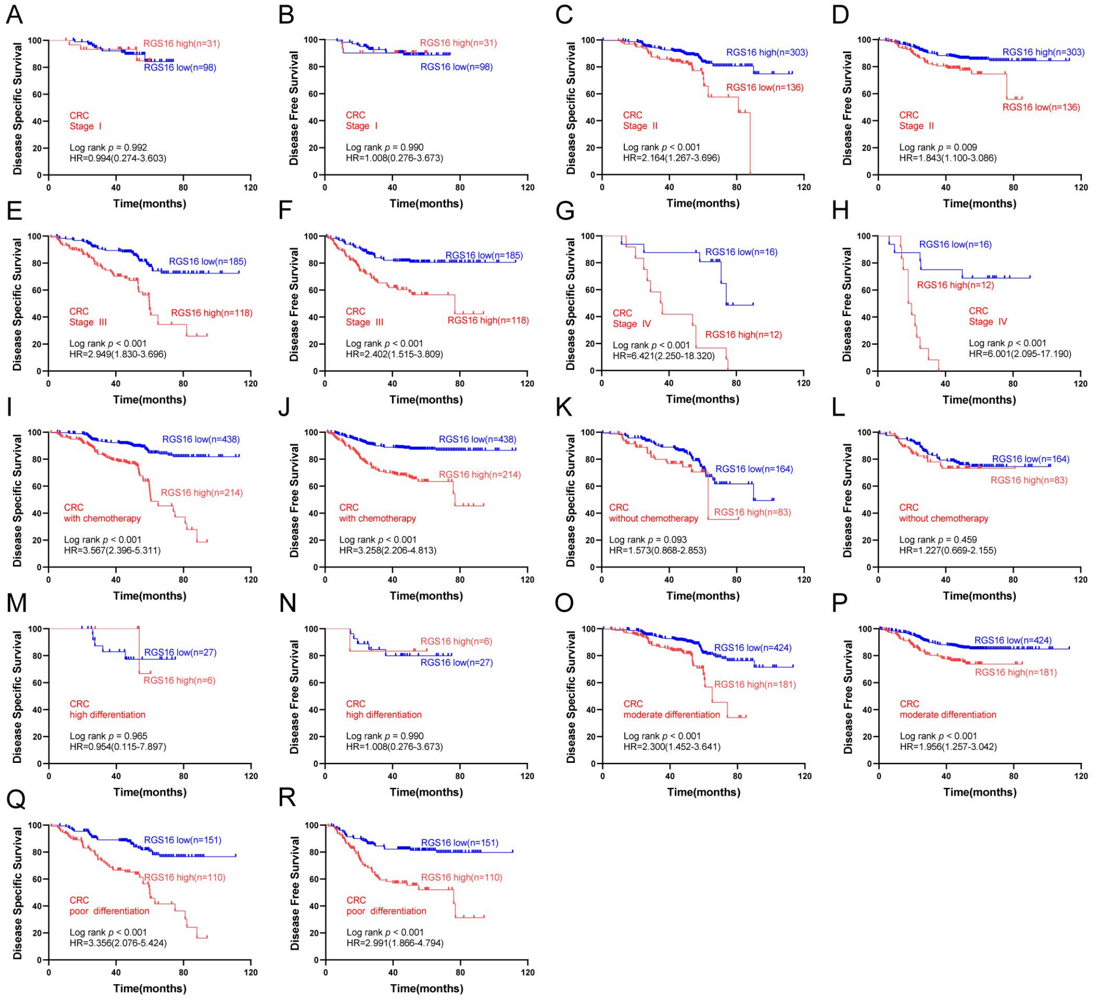

Supplementary Fig.S2 Kaplan-Meier survival curves for DSS and DFS in the subgroup according to RGS16 expression. A, B Kaplan-Meier curves showing no difference in the DSS(**A**) and DFS(**B**) of stage I patients separated based on the best cutoff value of RGS16 IHC scores. **C, D** Kaplan-Meier curves showing differences in the DSS(**C**) and DFS(**D**) of stage II patients separated based on the best cutoff value of RGS16 IHC scores. **E, F** Kaplan-Meier curves showing differences in the DSS(**E**) and DFS(**F**) of stage III patients separated based on the best cutoff value of RGS16 IHC scores. **G, H** Kaplan-Meier curves showing differences in the DSS(**C**) and DFS(**D**) of stage IV patients separated based on the best cutoff value of RGS16 IHC scores. **I, J** Kaplan-Meier curves showing differences in the DSS(**I**) and DFS(**J**) of patients with chemotherapy separated based on the best cutoff value of RGS16 IHC scores. **K, L** Kaplan-Meier curves showing no difference in the DSS(**K**) and DFS(**L**) of patients without chemotherapy separated based on the best cutoff value of RGS16 IHC scores. **M, N** Kaplan-Meier curves showing no difference in the DSS(**M**) and DFS(**N** of highly differentiated patients separated based on the best cutoff value of RGS16 IHC scores. **O, P** Kaplan-Meier curves showing differences in the DSS(**O**) and DFS(**P**) of moderately differentiated patients separated based on the best cutoff value of RGS16 IHC scores. **Q, R** Kaplan-Meier curves showing differences in the DSS(**Q**) and DFS(**R**) of poorly differentiated patients separated based on the best cutoff value of RGS16 IHC scores.

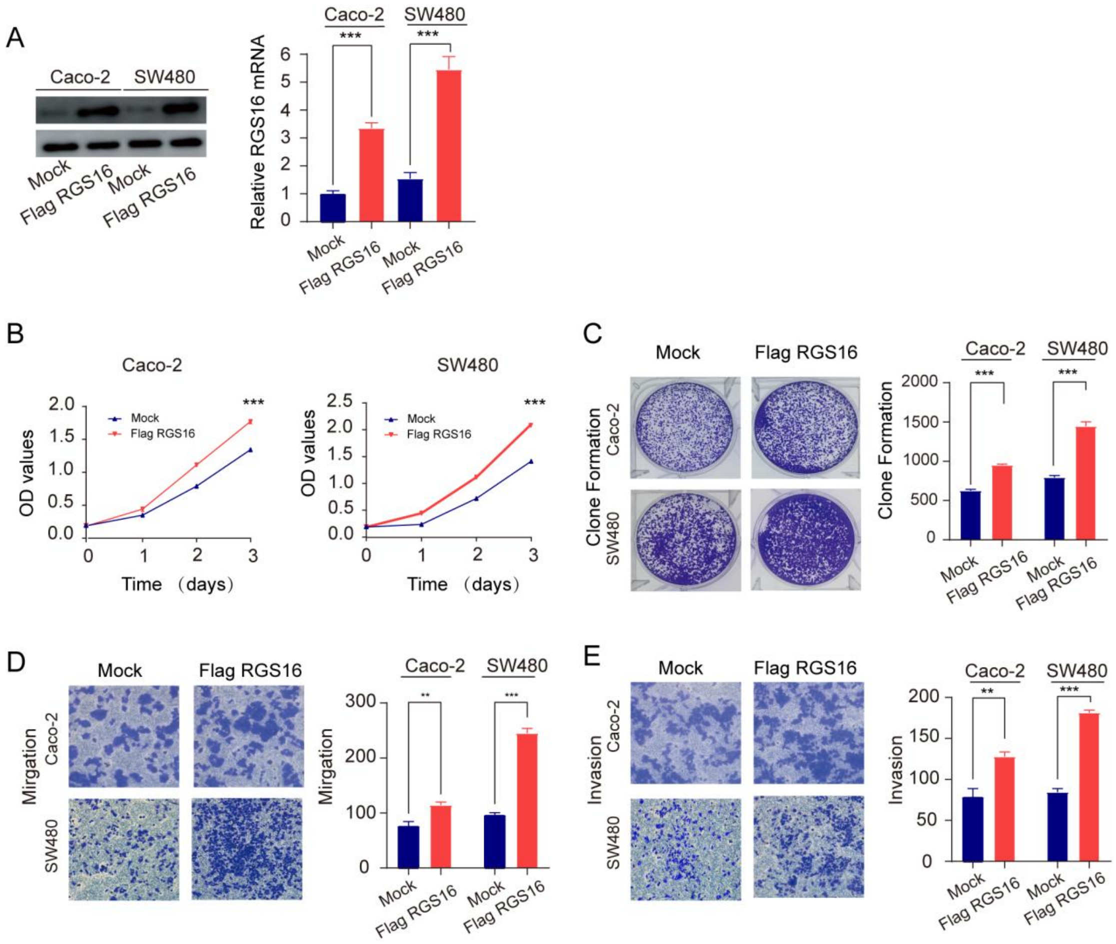

Supplementary Fig.S3 Overexpression of RGS16 promotes the progression of CRC cells in vitro. A, WB and qRT-PCR were used to measure the protein and mRNA concentrations of RGS16 in Caco-2 and SW480 cells that had been stably transfected with either a control (mock) or Flag RGS16 vector. **B,** Cell viability of Caco-2 and SW480 cells transfected with the mock or Flag RGS16 vector was determined using the CCK-8 assay at 1, 2, and 3 days to measure cell proliferation. **C,** Representative images of SW480 and Caco-2 cells after lentivirus infection were captured after 12 days of culture, and the number of cells was analyzed in a clonal growth formation experiment. **D, E** Migration(**D**) and invasion(**E**) of SW480 and Caco-2 infected cells were assessed using transwell membranes, as depicted in representative images. Scale bars = 100 μm. Quantitative analysis of migrated and invaded Caco-2 and SW480 cells was conducted. The data represent the mean ± SD of at least three independent experiments. The p-values were calculated by Student's t-test. **, and *** indicate that P is below 0.01 and 0.001, respectively.

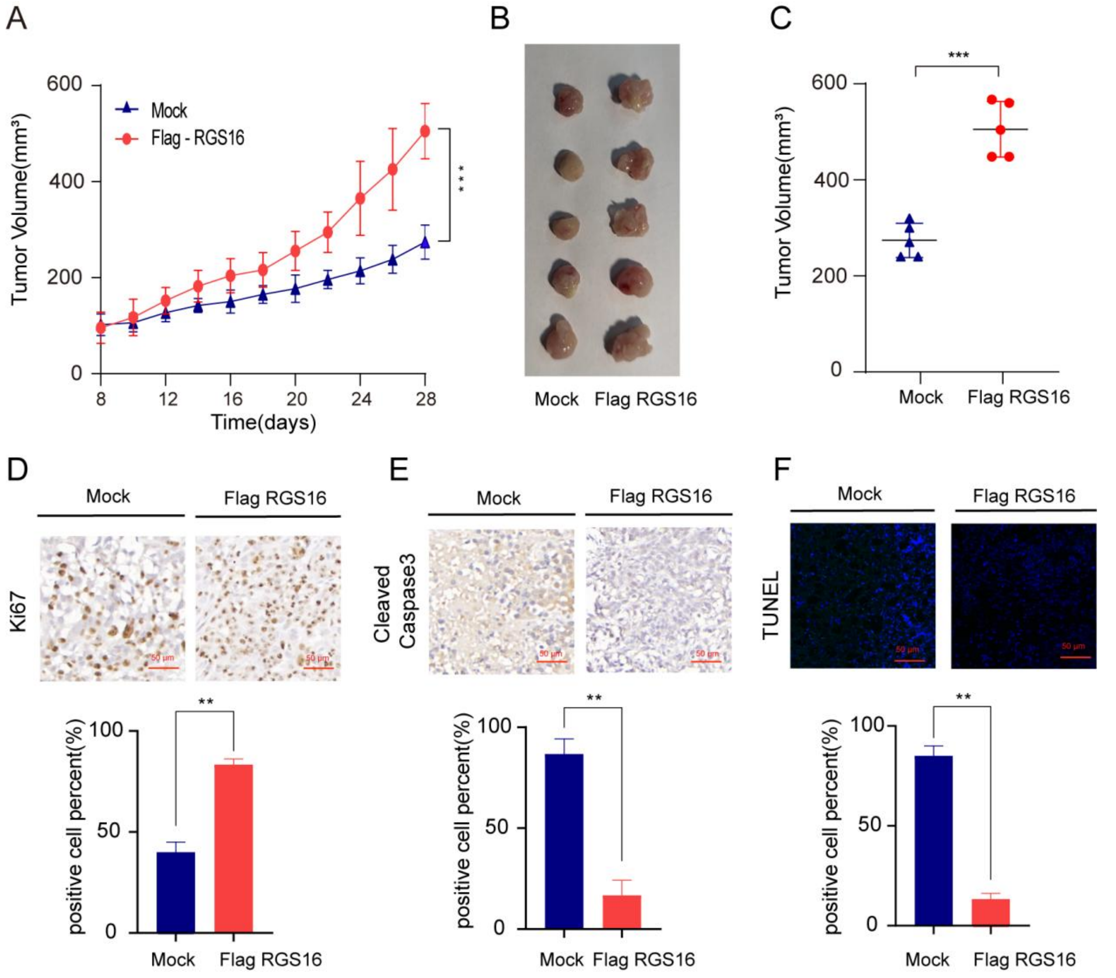

Supplementary Fig.S4 Overexpression of RGS16 promotes the growth of CRC xenografts in vivo. A Subcutaneous inoculation of mice was performed using Caco-2 cells that had been stably transfected with vectors, including mock and Flag RGS16. Tumor volume was measured as indicated when it reached 70-100 mm^3 (day 8) and calculated as $V = (width^2 \times length)/2$. **B** Volume difference of subcutaneous tumor xenografts after 4 weeks of feeding in nude mice. **C** The morphology of tumor xenografts from each nude mouse was photographed. **D, E** Immunohistochemistry staining of slices from xenografts was used to detect the expression of Ki67(**D**) and cleaved caspase 3(**E**). Scale bars, 50 μm. **F** TUNEL staining of tumor xenografts from the above two groups are shown. Scale bars, 50 μm. The p-values in panle were calculated by Student's t-test. **, and *** indicate that P is below 0.01 and 0.001, respectively. n = 5 mice/group.

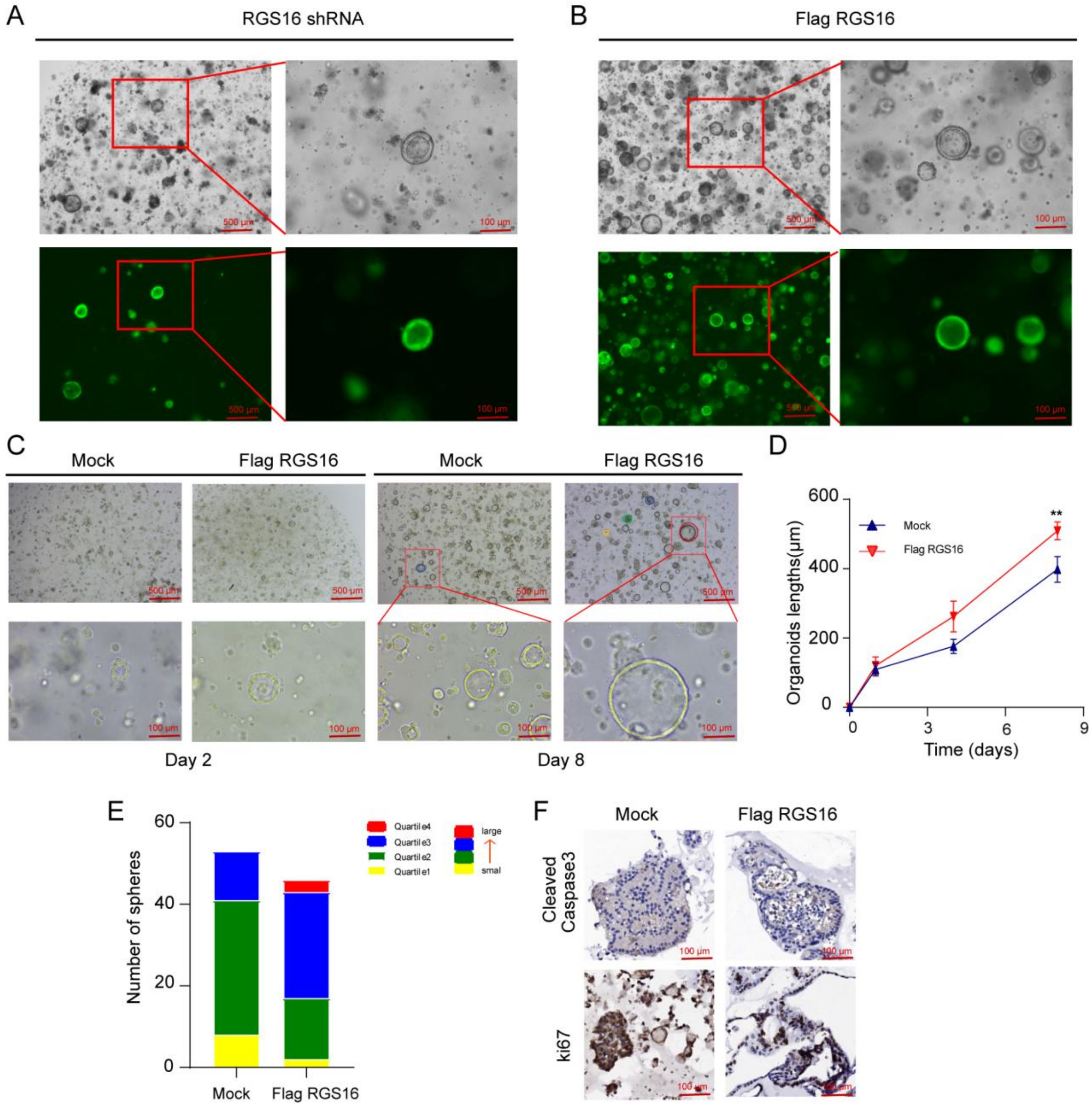

Supplementary Fig.S5 Overexpression of RGS16 facilitated apoptosis of tumor cell in CRC organoids. A, B Model and representative images of organoid for RGS16 infected with lentiviral constructs expressing RGS16 shRNA (**A**) or Flag RGS16 (**B**). **C, D** Images represented the CRC organoids after transfection with Ctrl and flag RGS16 vectors at Day2(**C**) and Day8(**D**). **E, F** CRC organoids length and shperes numbers of the above two groups was recorded. (**E**)The length of organoids in overexpression group was smaller than that in control group. (**F**)The proportion of smaller organoids in the overexpression group was also higher than that in the control group. **G, H** The representative graphs depicted the IHC staining illustrating Cleaved Caspase 3(**G**) and ki67(**H**) expression levels in both groups of organoids. The data represent the mean ± SD of at least three independent experiments. The p-values in panle C were calculated by Student's t-test. ***indicate that P is below 0.001.

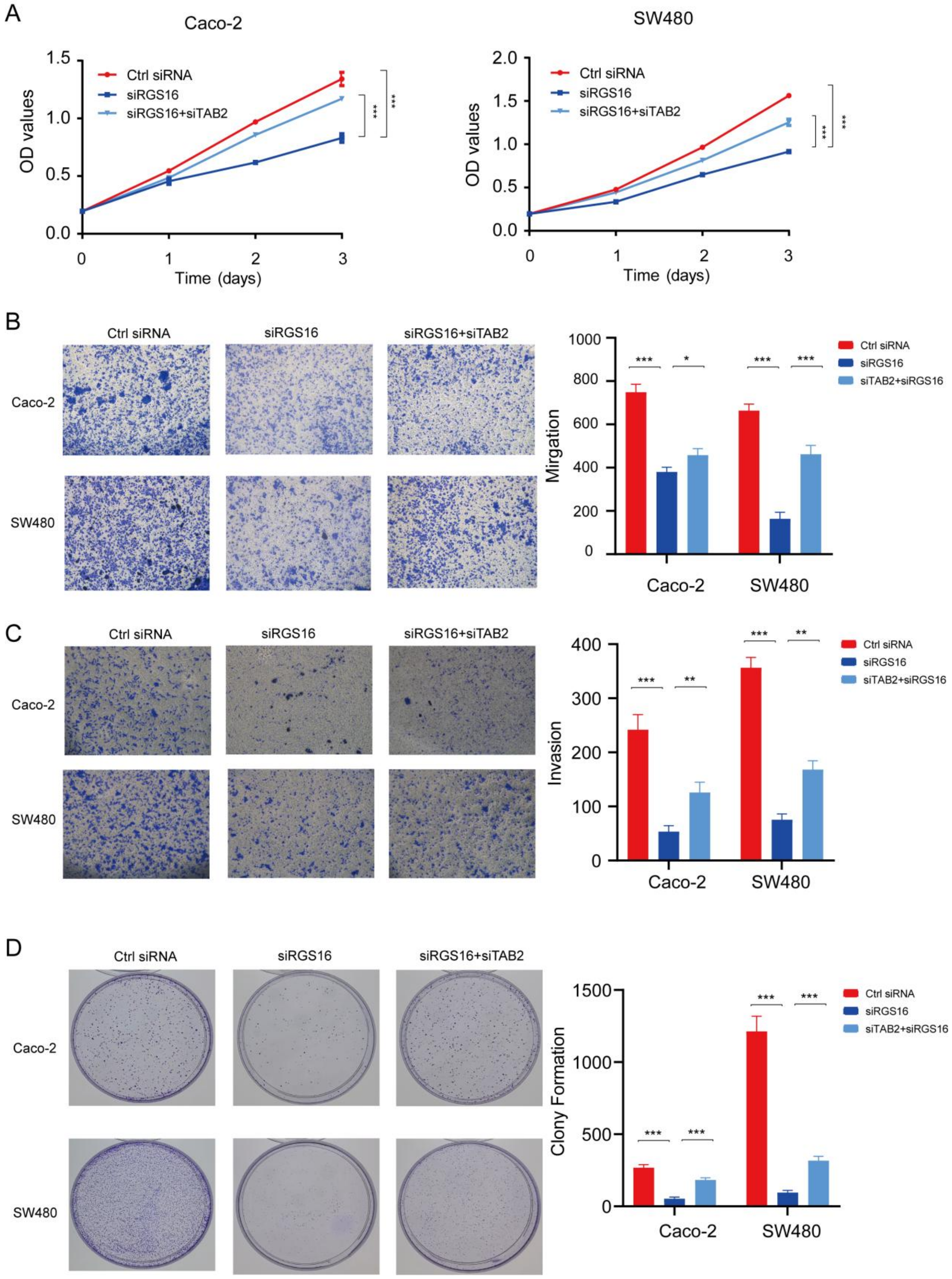

Supplementary Fig.S6 Inhibition of TAB2 reversed the anti-tumor effect of RGS16 knockdown. A Cell viability of Caco-2 and SW480 cells transfected with shRGS16 vector or TAB2 siRNAs was determined using the CCK-8 assay at 1, 2, and 3 days to measure cell proliferation. **B, C** Migration**(B)** and invasion**(C)** of SW480 and Caco-2 cells treated with shRGS16 vector or TAB2 siRNAs were assessed using transwell membranes, as depicted in representative images. Scale bars, 100 μm. Quantitative analysis of migrated and invaded Caco-2 and SW480 cells was conducted. **D** Representative images of SW480 and Caco-2 cells after lentivirus infection or treatment with TAB2 siRNAs were

captured after 12 days of culture, and the number of cells was analyzed in a clonal growth formation experiment. The data represent the mean ± SD of at least three independent experiments. The p-values in panles A, B, C and D were calculated by one-way ANOVA. *, **, and *** indicate that P is below 0.05, 0.01, and 0.001, respectively.
