# Supplementary material for: Regulator of G protein signaling 16 restrains apoptosis in colorectal cancer through disrupting TRAF6-TAB2-TAK1-JNK/p38 MAPK signaling
Source: Cell Death Dis. 2024 Jun 21;15(6):438. doi: 10.1038/s41419-024-06803-6 (PMC11192724; doi:10.1038/s41419-024-06803-6)
Supplement: Supplementary file 2 — supplementary material [file 41419_2024_6803_MOESM2_ESM.docx]

FIG 4A

|  |  |
| --- | --- |

FIG 4B

|  |  |
| --- | --- |

FIG 4H

|  |  |
| --- | --- |
|  |  |
|  |  |
|  |  |

FIG7D

|  |  |
| --- | --- |
|  |  |
|  |  |

FIG7E

|  |  |
| --- | --- |
|  |  |
|  |  |

FIG7F

|  |  |
| --- | --- |
|  |  |
|  |  |
|  |  |

FIG7G

FIG7H

FIG8A

|  |  |
| --- | --- |
|  |  |

FIG8B

|  |  |
| --- | --- |
|  |  |
|  |  |

FIGS2A

|  |  |
| --- | --- |
